# MGAviewer: a desktop visualization tool for analysis of metagenomics alignment data

**DOI:** 10.1093/bioinformatics/bts567

**Published:** 2012-10-08

**Authors:** Zhengwei Zhu, Beifang Niu, Jing Chen, Sitao Wu, Shulei Sun, Weizhong Li

**Affiliations:** Center for Research in Biological Systems, University of California San Diego, La Jolla, CA 92093, USA

## Abstract

**Summary:** Numerous metagenomics projects have produced tremendous amounts of sequencing data. Aligning these sequences to reference genomes is an essential analysis in metagenomics studies. Large-scale alignment data call for intuitive and efficient visualization tool. However, current tools such as various genome browsers are highly specialized to handle intraspecies mapping results. They are not suitable for alignment data in metagenomics, which are often interspecies alignments. We have developed a web browser-based desktop application for interactively visualizing alignment data of metagenomic sequences. This viewer is easy to use on all computer systems with modern web browsers and requires no software installation.

**Availability:** http://weizhongli-lab.org/mgaviewer

**Contact:** liwz@sdsc.edu

## 1 INTRODUCTION

The advances of Next Generation Sequencing technologies (Mardis, 2011) have promoted big waves of metagenomic projects in study of microbiomes under different environments such as ocean (Rusch *et al.*, 2007) and human body (Qin *et al.*, 2010). An essential step in metagenomic data analysis is to align the sequencing reads against the available microbial genomes.

Visualization is an intuitive way to analyze large-scale alignment data in genomic studies. There are many visualization tools available. Some are web browser-based such as UCSC genome browser (Dreszer *et al.*, 2012), LookSeq (Manske and Kwiatkowski, 2009) and JBrowse (Skinner *et al.*, 2009). Some are standalone programs such as Tablet (Milne *et al.*, 2010), GenomeView (Abeel *et al.*, 2012), MapView (Bao *et al.*, 2009), IGB (Nicol *et al.*, 2009), IGV (Robinson *et al.*, 2011), SamScope (Popendorf and Sakakibara, 2012) and so on.

However, these sophisticated visualization tools are specialized in handling intraspecies alignment results (i.e. query and reference are same species). They are not suitable for interspecies alignments from metagenomic datasets, where query and reference can be from different species. There are fundamental differences between intraspecies and interspecies alignments. The former only involves one reference genome and represent features like single nucleotide polymorphism and alternative splicing. But the latter involves multiple (often 10^3^) reference microbial genomes. To visualize interspecies alignments, a tool needs to show the wide range of alignment similarities (100% to as low as 50% for DNAs and 30% for proteins) and to handle thousands of reference genomes.

The Global Ocean Sampling study (Rusch *et al.*, 2007) first introduced fragment recruitment plots to illustrate the metagenomic alignment data. However, its underlying software is not available to the public.

Here, we present MetaGenomic Alignment Viewer (MGAviewer), a platform-independent web browser-based tool for visualizing alignment data. It does not rely on web server and relational database for image generation and data retrieval. It can be simply used as a standalone desktop program to analyze local data. It can also be included in a web server like other web-based genome browsers.

## 2 METHODS

**Fig. 1.**
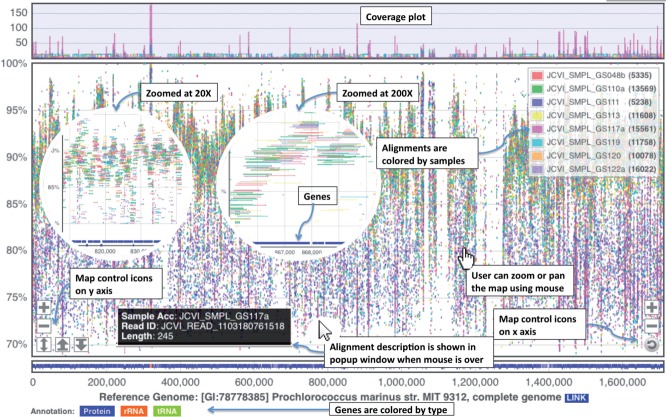
Screenshots of the MGAviewer visualization interface

The key component of this tool is a graphic interface with a 2D map that displays large amounts of alignments between metagenomic sequences from one or more samples and a reference genome (Fig. 1). Users can explore alignment data by interactively operating the 2D map in a similar way as in Google Maps.

MGAviewer is an HTML5 web application. It works in all major modern browsers, including Chrome, Firefox, Safari and Internet Explorer 9, without the need of installing any extra software or plugin. It uses jQuery (http://jquery.com/) as the base JavaScript library, and on top of which, a customized version of jQuery plotting plugin, Flot (http://code.google.com/p/flot/). We extended Flot to make it support drawing of fragments and annotation features. Above these, a site-specific JavaScript file (‘site.js’) is responsible for setting up plot parameters, placing and responding to additional controls and fetching data.

MGAviewer fetches alignment data from a user’s local computer or from a web server on demand via AJAX. It then draws the plot in an HTML5 Canvas element. Every time a user interaction event is triggered, e.g. zooming in/out, panning and resizing of the plot, the plot image is simply redrawn using data already loaded, unless additional data are required. This is in contrast to many other web-based genome browsers where plot images are generated on the server side and then retrieved by browser on demand; in MGAviewer a plot is drawn locally in browser. This results in no network traffic for most user operations and therefore dramatically improves the responsiveness of user interactions, especially on slow network.

Alignment data are stored in JSON (a lightweight data-interchange format used by JavaScript) formatted files, which contain alignment details including coordinate, sequence identity, name, e-value, etc. We provide scripts to generate JSON files from raw alignment results by BLAST (Altschul *et al.*, 1997) and FR-HIT (Niu *et al.*, 2011) and also from alignments in SAM format. These scripts need installation of BioPython package. Converters for other programs like BLAT (Kent, 2002) can be easily implemented.

MGAviewer can be used as standalone software by simply opening the directory that contains these JSON files, MGAviewer scripts and a master HTML file (see user’s guide for details). It can also be hosted on a web server. The plot itself can be embedded in any webpage.

## 3 RESULTS

MGAviewer has an interface for users to select one or more metagenomic samples and a reference from a list of reference genomes to generate the plot. The screenshots of MGAviewer are shown in Figure 1. The plot shows alignments from eight metagenomic samples to a reference genome. The *x*-axis is the genome coordinate, and *y*-axis is alignment identity (%). Alignments are coloured by sample and are represented as points or lines depending on zoom level. The bottom of the plot shows genes of the reference genome, and the top shows the genome coverage for each sample. Icons at left and right bottom corners are for zoom, resize and reset. Users can also zoom or pan the map by mouse. The inside circular images are zoomed views of the plot.

We tested MGAviewer on 1.5 million alignment datasets between >600 metagenomic samples from CAMERA (Sun *et al.*, 2011) and >2500 genomes from NCBI. MGAviewer provides real-time visualization for almost all these datasets except a few hundred very large datasets, which need extra several seconds for data loading and plotting. MGAviewer is already adopted by CAMERA project in its alignment resources, which will be described in a separate publication. MGAviewer can be used to analyze alignment data not only for prokaryotic species but also for viruses and small eukaryotic organisms.

*Funding*: This study was supported by Award R01HG005978 from the National Human Genome Research Institute and the Gordon and Betty Moore Foundation.

*Conflict of Interest*: none declared.

## References

[bts567-B1] Abeel T (2012). GenomeView: a next-generation genome browser. Nucleic Acids Res..

[bts567-B2] Altschul SF (1997). Gapped BLAST and PSI-BLAST: a new generation of protein database search
programs. Nucleic Acids Res..

[bts567-B3] Bao H (2009). MapView: visualization of short reads alignment on a desktop
computer. Bioinformatics.

[bts567-B4] Dreszer TR (2012). The UCSC genome browser database: extensions and updates
2011. Nucleic Acids Res..

[bts567-B5] Kent WJ (2002). BLAT–the BLAST-like alignment tool. Genome Res..

[bts567-B6] Manske HM, Kwiatkowski DP (2009). LookSeq: a browser-based viewer for deep sequencing data. Genome Res..

[bts567-B7] Mardis ER (2011). A decade’s perspective on DNA sequencing technology. Nature.

[bts567-B8] Milne I (2010). Tablet–next generation sequence assembly
visualization. Bioinformatics.

[bts567-B9] Nicol JW (2009). The integrated genome browser: free software for distribution and
exploration of genome-scale datasets. Bioinformatics.

[bts567-B10] Niu B (2011). FR-HIT, a very fast program to recruit metagenomic reads to homologous
reference genomes. Bioinformatics.

[bts567-B11] Popendorf K, Sakakibara Y (2012). SAMSCOPE: an openGL-based real-time interactive scale-free SAM
viewer. Bioinformatics.

[bts567-B12] Qin J (2010). A human gut microbial gene catalogue established by metagenomic
sequencing. Nature.

[bts567-B13] Robinson JT (2011). Integrative genomics viewer. Nat. Biotechnol..

[bts567-B14] Rusch DB (2007). The sorcerer II Global Ocean Sampling expedition: northwest Atlantic
through eastern tropical Pacific. PLoS Biol..

[bts567-B15] Skinner ME (2009). JBrowse: a next-generation genome browser. Genome Res..

[bts567-B16] Sun S (2011). Community cyberinfrastructure for advanced microbial ecology research and
analysis: the CAMERA resource. Nucleic Acids Res..

